# Disproportionately raised risk of adverse outcomes in patients with COPD and comorbid type 2 diabetes or depression: Swedish register-based cohort study

**DOI:** 10.1186/s12931-025-03160-6

**Published:** 2025-03-05

**Authors:** Carolina Smith, Mikael Hasselgren, Hanna Sandelowsky, Björn Ställberg, Ayako Hiyoshi, Scott Montgomery

**Affiliations:** 1 Clinical Epidemiology and Biostatistics, School of Medical Sciences, Faculty of Medicine and Health, Örebro University, Örebro, Sweden; 2 Centre for Clinical Research and Education, Region Värmland, Karlstad, Sweden; 3 School of Medical Sciences, Faculty of Medicine and Health, Örebro University, Örebro, Sweden; 4 Department of Neurobiology, Care Sciences and Society, Division of Family Medicine and Primary Care, Karolinska Institutet, Stockholm, Sweden; 5 Academic Primary Health Care Centre, Region Stockholm, Stockholm, Sweden; 6 Department of Public Health and Caring Sciences, Family Medicine and Preventive Medicine, Uppsala University, Uppsala, Sweden; 7 Department of Public Health Sciences, Stockholm University, Stockholm, Sweden; 8 Clinical Epidemiology Division, Department of Medicine, Karolinska Institutet, Solna, Stockholm, Sweden; 9 Department of Epidemiology and Public Health, University College London, London, UK

**Keywords:** COPD, Type 2 diabetes, Depression, Cardiovascular disease, Mortality

## Abstract

**Background:**

We aimed to examine if patients with COPD and comorbid type 2 diabetes, or COPD with comorbid depression or anxiety, had disproportionally raised excess risks of subsequent cardiovascular disease and mortality.

**Methods:**

This general population-based cohort study used data from Swedish national registers, with follow-up during 2005–2018. Cox regression estimated risks of cardiovascular disease or mortality, producing hazard ratios (HR) with (95% confidence intervals). Interaction testing quantified disproportionally increased excess risks.

**Results:**

Among 5,624,306 individuals, 332,549 had a COPD diagnosis. Compared with individuals who did not have COPD or type 2 diabetes, all-cause mortality risk was higher for individuals who had either COPD or type 2 diabetes, with HR 2.68 (2.66–2.69) and 1.70 (1.69–1.71), respectively. Having both conditions produced an HR of 3.72 (3.68–3.76). Among cardiovascular outcomes, the highest risks were found for chronic heart failure: COPD only, HR 2.87 (2.84–2.90); type 2 diabetes only, 1.86 (1.84–1.88); and both, 4.55 (4.46–4.64). Having both COPD and type 2 diabetes was associated with disproportionally higher excess risks than expected from the sum of the individual diseases, except for cerebrovascular disease or ischemic heart disease. For COPD and depression/anxiety, all-cause mortality risk was associated with COPD only, HR 2.74 (2.72–2.76); depression/anxiety only, 2.39 (2.38–2.40); and both 4.72 (4.68–4.75). Chronic heart failure was associated with COPD only, HR 2.74 (2.71–2.78); depression/anxiety only, 1.31 (1.30–1.32); and both, 3.45 (3.40–3.50). This disease combination was associated with disproportionally higher excess risks than expected, except for atrial fibrillation.

**Conclusions:**

Type 2 diabetes or depression/anxiety in COPD patients were associated with disproportionally excess risks for cardiovascular disease and mortality. It is important for clinicians to be aware of these greater than expected risks, to prevent further cardiovascular morbidity and mortality.

**Supplementary Information:**

The online version contains supplementary material available at 10.1186/s12931-025-03160-6.

## Introduction

Most patients with chronic obstructive pulmonary disease (COPD) also have other chronic disorders [1, 2]. Some diseases (or their treatments) may interact, resulting in disproportionally adverse outcomes that are greater in magnitude than the sum of risks from the two diseases. It is therefore of value to identify such combinations, so that specific risk mechanisms can be investigated, and more effective management strategies can be developed for primary care where management of multimorbidity is increasingly common. Type 2 diabetes, depression and anxiety are some of the most common comorbid diseases in patients with COPD in primary care [1], so it may be of value to identify whether COPD patients with these specific comorbid diseases are at a disproportionally raised risk of adverse outcomes.

Patients with COPD and comorbid type 2 diabetes may have an increased risk of hospital admission and mortality [3, 4]. In a previous study, we found some evidence that management of COPD in primary care settings may be less prioritised in patients that also have diabetes, possibly because care for diabetes is prioritised [5]. This is consistent with the findings of a Danish study where annual primary care consultations for COPD follow-up were less frequent among patients with both COPD and diabetes than patients with only COPD [6]. Anxiety and depression frequently co-occur in patients with COPD, and are more common than in the general population [7]. Comorbid depression or anxiety have been associated with an increased risk of COPD exacerbations and mortality [7–9]. Despite this, these conditions are often unrecognised and untreated in patients with COPD [7]. Many patients have persistent depressive symptoms for years [10]. COPD, type 2 diabetes, and depression are all risk factors for cardiovascular disease [11–13], so it is important to quantify any disproportionally excess risk arising from specific disease combinations to identify population subgroups which can benefit from enhanced interventions.

We hypothesized that patients with the combinations of COPD and type 2 diabetes, or COPD with a history of depression and/or anxiety, represent risks for adverse outcomes that are of higher magnitude than the sum of the risks associated with the individual diseases. The aim of the study was to examine the extent to which these combinations are associated with disproportionally raised risks for specific cardiovascular diagnoses and mortality.

## Methods

### Study population and design

All residents in Sweden between 1st July 2005 and 31st of December 2018 who were at least 40 years of age by 31st of December 2018 were identified in the Swedish Total Population Register. The register contains information on birth, death, and migration of all Swedish residents.

We used a cohort study design with follow-up time starting on 1st July 2005 or at age 40 years, which ever occurred last. Follow-up ended on the date of emigration, death, age 100 years, first diagnosis of the outcome or 31st December 2018, whichever came first. We excluded individuals who immigrated to Sweden after 1997 or had missing data for covariates.

### Exposures

We modelled COPD, type 2 diabetes, and depression or anxiety as time-varying exposure variables, which changed status from “no” to “yes” on the first day that relevant information was recorded in the National Patient Register or the National Prescribed Drug Register. The Patient Register has complete coverage of inpatient care from 1987 and outpatient specialist care from 2001 [14]. Data from primary care is not included in the register. We used this register to identify diagnoses from 1997 and onwards. Both primary and secondary diagnoses were used for identification. The Prescribed Drug Register was used to identify primary care patients with the target diseases that had not been diagnosed in secondary care (specialist visits or hospital admission), as it provided information on prescribed drugs dispensed in pharmacies from 2005 and onwards [15].

### COPD

We defined COPD from the first diagnosis (ICD-10 code J44) or the first day a prescription of long-acting muscarinic antagonists (LAMA) with ATC-code R03BB was dispensed at a pharmacy. Since LAMA also can be prescribed for patients with asthma, we excluded individuals with a dispensed prescription of LAMA before 45 years of age, or prescription of leukotriene receptor antagonists (R03DC), or with a primary diagnosis of asthma (J45-46).

### Type 2 diabetes

We defined type 2 diabetes from the first diagnosis (E11) or the first day a blood glucose lowering drug (A10B) was dispensed at a pharmacy. We also included dispensed prescriptions of insulin (A10A), in individuals without a diagnosis of any other type of diabetes (E10, E12-14). Women younger than 45 years of age at first dispensed prescription of insulin were not identified as having type 2 diabetes (*n* = 901) due to then possibility of gestational diabetes treatment.

### Depression/anxiety

We defined depression/anxiety from the earliest date of diagnosis (F32-33, or F41) or the first day that antidepressant drugs (N06A) were dispensed from a pharmacy with an appropriate defined daily dose (DDD), and treatment duration exceeded 180 days within a 365-day period.

### Outcomes

The outcomes are incident cardiovascular diseases and mortality (cause-specific and all-cause).

### Cardiovascular diseases

We identified the incidence of cardiovascular diseases through the Patient Register. The earliest date with a record of relevant ICD-10 codes, as primary or secondary diagnoses, was defined as onset date. We used ICD-10 codes I48 for atrial fibrillation, I50 for chronic heart failure, I60-69 for cerebrovascular disease, I20-22 and I24-25 for ischaemic heart disease, and I70-73 for peripheral arterial disease.

### Cause-specific and all-cause mortality

In addition to all-cause mortality, deaths from respiratory (ICD-10 code J) and cardiovascular causes (ICD-10 code I) were identified using both underlying and contributory causes of death recorded in the Cause of Death Register [16]. These outcomes were not mutually exclusive, as an individual could have both a respiratory and a cardiovascular cause of death.

### Covariates

We adjusted for the highest attained level of education by age 40 years (or in 1990 for those already older than 40 years) as a marker of socioeconomic characteristics, using data from the Longitudinal Integrated Database for Health Insurance and Labour Market Studies, annually compiled since 1990. Data on county of residence in 2005, or when an individual attained age 40 years and entered the study, were obtained from the Total Population Register.

### Statistical analysis

For descriptive data, categorical variables were described using frequencies and percentages. Continuous variables were described using median and range.

We used Cox regression to assess the risk of the outcomes (incident disease or mortality). Both COPD and the comorbid diseases were modelled as time-varying dichotomous covariates. Age was the underlying timescale. Each outcome was analysed separately. Hazard ratios (HR) with 95% confidence intervals (CI) were calculated. The proportional hazards assumption was assessed using Schoenfeld residual tests and plots. Although we observed statistically significant Ρ-values for the Schoenfeld residual test for most outcomes, this seemed to be due to the large sample size, as when smaller random samples (10,000 individuals) were assessed, the tests were not statistically significant. Thus, we present results assuming proportionality. Two analyses were conducted: one adjusting only for age (using it as the time scale), and the other further adjusting for sex, level of attained education, county of residence, and year of entry in the study.

To assess whether patients with the combinations of COPD with comorbid type 2 diabetes or COPD with depression/anxiety have disproportionally worse outcomes, we examined interactions between COPD and the comorbid disease on an additive scale, calculating the relative excess risk due to interaction (RERI). Additive interactions are arguably the most relevant measure in public health [17, 18]. The additive interaction is based on the sum of the individual disease effects and tests whether the combined effect of two exposures, for example COPD and diabetes, is larger or smaller than the *sum* of the individual effects of the two exposures [19, 20]. The RERI is the risk difference formulated using HRs (RERI = HR_COPD + diabetes_– HR_COPD_– HR_diabetes_ + 1). A positive value indicates that the combined risk is larger than the sum of individual effects, a negative value that the combined risk is smaller, and zero that the combined risk equals the sum of risks.

As a sensitivity analysis, we repeated all analyses with COPD defined as only those who had their first diagnosis of COPD identified through the Prescribed Drug Register to examine patients principally treated in primary care.

Data management and statistical analysis were performed using Stata version 18.

## Results

### Population characteristics

From among the 5,738,096 eligible individuals, we excluded those who had missing data for county of residence (*n* = 47,385, 0.8%) or attained level of education (*n* = 66,405, 1.2%). We analysed data for the remaining 5,624,306 individuals, where 51.2% were female, and 332,549 had a diagnosis of COPD, see Table 1. Median age at the start of study period was 53 years (range 27–100 years).

**Table 1:** Study population characteristics

	Non-COPD	COPD*
Number of individuals, n	5 291 757	332 549
Women, n (%)	2 703 982 (51.1)	178 110 (53.6)
Median age at start of study period, years (range)	52 (27–100)	66 (27–96)
Type 2 diabetes, n (%)	554 510 (10.5)	65 077 (19.6)
Depression/anxiety, n (%)	1 625 232 (30.7)	160 717 (48.3)

*Diagnosis sometime during 1997–2018

### COPD and type 2 diabetes

Compared with people without these diseases, there was an increased risk among patients with type 2 diabetes or COPD for cardiovascular disease and mortality (Table 2). The highest magnitude risks were found for those with both COPD and type 2 diabetes, particularly for chronic heart failure and mortality.

The combination of COPD and type 2 diabetes was associated with an increased outcome risk of greater magnitude than would be expected from the sum of the risks of the individual diseases for most outcomes, as indicated by positive values of the interaction estimates (shown in Table 2). The highest excess risks were found for chronic heart failure and respiratory causes of death. For ischemic heart disease and cerebrovascular disease, we found no evidence of additional risk for those with both COPD and type 2 diabetes.

**Table 2:** COPD and type 2 diabetes

Outcome	No of outcome events/individuals	Unadjusted	Adjusted	
	*n*	HR (95% CI)	HR (95% CI)	Additive interaction (95% CI)
Cardiovascular disease				
**Atrial fibrillation**	453 280/5 464 764			
No diabetes or COPD		Reference	Reference	0.15 (0.01, 0.20)
Type 2 diabetes		1.42 (1.40, 1.43)	1.35 (1.34, 1.36)	
COPD		1.64 (1.63, 1.66)	1.63 (1.61, 1.64)	
COPD and type 2 diabetes		2.25 (2.19, 2.30)	2.13 (2.08, 2.18)	
**Cerebrovascular disease**	356 848/5 481 249			
No diabetes or COPD		Reference	Reference	<-0.01 (-0.06, 0.05)
Type 2 diabetes		1.55 (1.53, 1.56)	1.48 (1.46, 1.49)	
COPD		1.43 (1.42, 1.45)	1.41 (1.39, 1.43)	
COPD and type 2 diabetes		1.99 (1.93, 2.04)	1.88 (1.83, 1.93)	
**Chronic heart failure**	383 601/5 515 858			
No diabetes or COPD		Reference	Reference	0.82 (0.73, 0.91)
Type 2 diabetes		1.97 (1.95, 1.98)	1.86 (1.84, 1.88)	
COPD		2.95 (2.92, 2.98)	2.87 (2.84, 2.90)	
COPD and type 2 diabetes		4.90 (4.81, 5.00)	4.55 (4.46, 4.64)	
**Ischemic heart disease**	429 938/5 355 693			
No diabetes or COPD		Reference	Reference	-0.02 (-0.09, 0.05)
Type 2 diabetes		1.82 (1.80, 1.84)	1.69 (1.67, 1.70)	
COPD		1.85 (1.83, 1.88)	1.83 (1.81, 1.85)	
COPD and type 2 diabetes		2.68 (2.61, 2.75)	2.50 (2.43, 2.56)	
**Peripheral arterial disease**	176 588/5 569 229			
No diabetes or COPD		Reference	Reference	0.14 (0.03, 0.25)
Type 2 diabetes		1.86 (1.84, 1.89)	1.73 (1.71, 1.75)	
COPD		2.64 (2.60, 2.69)	2.58 (2.54, 2.62)	
COPD and type 2 diabetes		3.74 (3.63, 3.85)	3.45 (3.35, 3.56)	
***Mortality***				
**All-cause mortality**	1 078 751/5 624 306			
No diabetes or COPD		Reference	Reference	0.34 (0.30, 0.39)
Type 2 diabetes		1.78 (1.77, 1.79)	1.70 (1.69, 1.71)	
COPD		2.74 (2.72, 2.75)	2.68 (2.66, 2.69)	
COPD and type 2 diabetes		3.93 (3.89, 3.98)	3.72 (3.68, 3.76)	
**Cardiovascular death**	655 441/5 624 306			
No diabetes or COPD		Reference	Reference	0.75 (0.69, 0.82)
Type 2 diabetes		1.97 (1.96, 1.98)	1.88 (1.86, 1.89)	
COPD		2.79 (2.77, 2.81)	2.72 (2.70, 2.74)	
COPD and type 2 diabetes		4.65 (4.59, 4.71)	4.35 (4.30, 4.41)	
**Respiratory death**	252 179/5 624 306			
No diabetes or COPD		Reference	Reference	1.59 (1.41, 1.76)
Type 2 diabetes		1.71 (1.69, 1.73)	1.62 (1.60, 1.65)	
COPD		8.80 (8.72, 8.89)	8.55 (8.47, 8.63)	
COPD and type 2 diabetes		11.56 (11.38, 11.75)	10.76 (10.59, 10.93)	

Cox regression with COPD and type 2 diabetes diagnoses being modelled as time-varying covariates. The adjusted HRs were obtained by controlling for sex, highest attained level of education, county of residence, and entrance year in the study. The additive interactions were calculated as the relative excess risk due to interaction (RERI)

*Abbreviations*: HR hazard ratio; CI confidence interval

### COPD and depression/anxiety

Compared with individuals with neither disease, patients with depression/anxiety or COPD had an increased risk for all outcomes (Table 3). The highest magnitude risk was seen among those with both diseases, particularly for chronic heart failure and mortality.

The combination of COPD and depression/anxiety was associated with a disproportionally higher excess risk for all cardiovascular diseases except atrial fibrillation. The highest magnitude risk was found for chronic heart failure. Having both conditions was associated with disproportionally higher magnitude risks for all-cause and cause-specific mortality, particularly due to respiratory disease.

**Table 3:** COPD and depression/anxiety

Outcome	No of outcome events/individuals	Unadjusted	Adjusted	
	*N*	HR (95% CI)	HR (95% CI)	Additive interaction (95% CI)
Cardiovascular disease				
**Atrial fibrillation**	453 280/5 464 764			
No COPD or depression/anxiety		Reference	Reference	<-0.01 (-0.04, 0.04)
Depression/anxiety		0.97 (0.96, 0.97)	1.04 (1.03, 1.05)	
COPD		1.68 (1.65, 1.70)	1.63 (1.61, 1.65)	
COPD and depression/anxiety		1.57 (1.55, 1.60)	1.67 (1.64, 1.70)	
**Cerebrovascular disease**	356 848/5 481 249			
No COPD or depression/anxiety		Reference	Reference	0.09 (0.05, 0.13)
Depression/anxiety		1.28 (1.27, 1.29)	1.37 (1.36, 1.38)	
COPD		1.39 (1.37, 1.41)	1.35 (1.33, 1.37)	
COPD and depression/anxiety		1.73 (1.70, 1.76)	1.81 (1.77, 1.84)	
**Chronic heart failure**	383 601/5 515 858			
No COPD or depression/anxiety		Reference	Reference	0.40 (0.34, 0.45)
Depression/anxiety		1.21 (1.20, 1.22)	1.31 (1.30, 1.32)	
COPD		2.87 (2.83, 2.90)	2.74 (2.71, 2.78)	
COPD and depression/anxiety		3.29 (3.25, 3.34)	3.45 (3.40, 3.50)	
**Ischemic heart disease**	429 938/5 355 693			
No COPD or depression/anxiety		Reference	Reference	0.16 (0.12, 0.20)
Depression/anxiety		1.08 (1.07, 1.08)	1.20 (1.19, 1.21)	
COPD		1.79 (1.77, 1.82)	1.73 (1.71, 1.76)	
COPD and depression/anxiety		1.91 (1.88, 1.95)	2.09 (2.06, 2.13)	
**Peripheral arterial disease**	176 588/5 569 229			
No COPD or depression/anxiety		Reference	Reference	0.16 (0.08, 0.24)
Depression/anxiety		1.21 (1.19, 1.22)	1.33 (1.31, 1.34)	
COPD		2.60 (2.56, 2.65)	2.49 (2.44, 2.53)	
COPD and depression/anxiety		2.78 (2.72, 2.85)	2.98 (2.91, 3.05)	
***Mortality***				
**All-cause mortality**	1 078 751/5 624 306			
No COPD or depression/anxiety		Reference	Reference	0.59 (0.55, 0.63)
Depression/anxiety		2.22 (2.21, 2.23)	2.39 (2.38, 2.40)	
COPD		2.86 (2.83, 2.88)	2.74 (2.72, 2.76)	
COPD and depression/anxiety		4.53 (4.49, 4.56)	4.72 (4.68, 4.75)	
**Cardiovascular death**	655 441/5 624 306			
No COPD or depression/anxiety		Reference	Reference	0.73 (0.68, 0.78)
Depression/anxiety		2.11 (2.09, 2.12)	2.28 (2.27, 2.29)	
COPD		2.90 (2.87, 2.93)	2.77 (2.74, 2.79)	
COPD and depression/anxiety		4.56 (4.52, 4.60)	4.77 (4.73, 4.82)	
**Respiratory death**	252 179/5 624 306			
No COPD or depression/anxiety		Reference	Reference	5.68 (5.50, 5.85)
Depression/anxiety		2.43 (2.41, 2.46)	2.66 (2.63, 2.68)	
COPD		9.24 (9.14, 9.35)	8.78 (8.68, 8.88)	
COPD and depression/anxiety		15.34 (15.17, 15.52)	16.11 (15.93, 16.30)	

Cox regression with COPD and depression/anxiety diagnoses being modelled as time-varying covariates. The adjusted HRs were obtained by controlling for sex, highest attained level of education, county of residence, and entrance year in the study. The additive interactions were calculated as the relative excess risk due to interaction (RERI)

*Abbreviations*: HR hazard ratio; CI confidence interval

### Sensitivity analysis

When the patients with COPD was limited to those with COPD first identified using prescribed medication, to identify a predominantly primary care population, the results were consistent with the main analysis, but with lower magnitude risk estimates, see Supplemental Tables 1 and 2. For COPD and type 2 diabetes, the excess risk for peripheral arterial disease was no longer statistically significant, while the risk for cerebrovascular disease was statistically significantly lower.

## Discussion

In this large general population-based study, we found that individuals with COPD and coexisting type 2 diabetes tended to have an excess risk of both subsequent cardiovascular disease (except ischemic heart disease and cerebrovascular disease) and mortality that was greater than the sum of the individual disease risks. Having both COPD and depression/anxiety was also associated with a disproportionally excess risk of cardiovascular disease (except atrial fibrillation) and mortality. As identification of COPD, type 2 diabetes, and depression/anxiety included prescribed medication dispensed at pharmacies across Sweden, this study not only captured individuals treated through outpatient consultation or hospital admission, but also individuals with pharmacological treatment in primary care.

COPD, type 2 diabetes, and depression/anxiety are mainly managed in primary care where consultation time may be scarce, and many conditions need to be managed during a single consultation [21]. Thus, a certain prioritisation among diseases is likely to be made, by both doctor and patient. While management of COPD may be de-prioritised in patients with type 2 diabetes in primary care [5, 6], our findings indicate that clinicians should be vigilant when patients have both diseases, to prevent further morbidity and increased mortality. It would be beneficial to identify the pathways leading to this disproportionally increased risk and possibly develop tailored management strategies that deal with the combination, rather than just the individual diseases. Also, we found higher risk estimates for most outcomes in patients with only COPD than in patients with only diabetes, which highlights the need for increased priority of COPD management.

Type 2 diabetes, COPD, and arteriosclerosis have shared risk factors such as older age, smoking, and also pathogenic factors, such as systemic inflammation [22]. In a recent Canadian study, patients with COPD were 25% more likely to have a major cardiovascular event compared with people without COPD, which is similar to the excess risk of cardiovascular events in patients with diabetes [23]. A study from the UK found that individuals with both type 2 diabetes and COPD had higher all-cause and respiratory mortality than individuals with only diabetes [24]. This is consistent with our findings, where we found the risk of mortality from respiratory and cardiovascular causes as well as all-cause mortality to be larger than the sum of the risks associated with COPD and diabetes. For cardiovascular morbidity we also found disproportionally high magnitude excess risks for individuals with both COPD and type 2 diabetes, mainly for chronic heart failure, peripheral arterial disease, and atrial fibrillation, but not for ischaemic heart disease and cerebrovascular disease. Both COPD and type 2 diabetes have been associated with increased risk of chronic heart failure and peripheral arterial disease [11, 25, 26], and our findings suggest that the combined risk is larger than the sum of risks associated with the individual diseases. Our findings may be an indication of a potential biological interaction between COPD and type 2 diabetes, although other factors, such as de-prioritisation of treatment, may also be relevant.

Depression is a known risk factor for cardiovascular disease, sharing pathogenic factors such as inflammation and stress [13]. Mechanisms connecting depression and COPD remain unclear, but systemic inflammation and associations with smoking have been suggested [27]. Patients with depression are more likely to smoke and to have poor adherence to treatment [28, 29]. Except for atrial fibrillation, we found excess risks for all cardiovascular diseases and mortality for individuals with both COPD and depression/anxiety. The combined risks for those with both diseases were larger than the sum of the individual diseases, especially for death due to respiratory disease. Our findings may be an indication of a biological interaction between the diseases or a manifestation of the vicious circle of COPD and depression/anxiety, leading to further deterioration [30].

There has recently been a call for a more active approach towards identification of cardiovascular disease and risk in patients with COPD, and for COPD in patients with cardiovascular disease [31]. Our findings indicate that this may be of particular importance in patients with comorbid type 2 diabetes or depression/anxiety. It is possible that any comorbidity with COPD represents a non-specific marker of poorer health. However, our analysis was adjusted for potential confounding factors, and the combinations of COPD with type 2 diabetes and depression/anxiety were not associated with all the outcome diseases, suggesting that there is a disease-specific risk, and that it may be possible to develop disease combination-specific interventions. As most of these patients are managed in primary care, a focus should be on improving primary care management strategies for specific disease combinations.

Strengths of this study include the large sample size and use of prospectively recorded register data including all people in Sweden. The Patient Register has high coverage and validity for most hospital diagnoses [14]. The completeness of the Cause of Death Register is high [32]. The Prescribed Drug Register will have identified 100% of patients that collected prescribed medication from a pharmacy, thus producing results relevant to the primary care population treated pharmacologically [15].

This study also has some potential limitations. Identification of diagnoses through the Patient Register, excluded patients solely managed in primary care, however, the use of the Prescribed Drug Register and dispensed prescriptions of disease-specific drugs allowed us to also identify patients who had not received hospital care but been managed, and pharmaceutically treated, in primary care. In Sweden, most patients with COPD are managed in primary care [33]. Therefore, we performed a sensitivity analysis, including only COPD-diagnoses first identified by the Prescribed Drug Register. The results were consistent with the main results, but of lower magnitude, corresponding to a presumably somewhat healthier primary care population. For type 2 diabetes and COPD, there was no excess risk for ischemic heart disease, cerebrovascular disease, and peripheral arterial disease.

Some patients with asthma, with late-onset or use of LAMA, may have been misclassified as having COPD, but this would only be a small number of patients, thus unlikely to have affected the results notably. Individuals with diabetes were limited to those who were pharmaceutically treated for diabetes or had the diagnosis in outpatient or inpatient care. Individuals who were treated only with diet were not captured, possibly leading our definition of diabetes to include individuals with more severe diabetes. For depression/anxiety, we used uninterrupted prescriptions of antidepressant drugs for at least six months, likely identifying a larger proportion of patients treated in primary care. Our definition may also include some patients with a history of depression/anxiety, possibly without long-term persistent symptoms. Choosing the earliest date of diagnosis or dispensed prescription as the incident date is arbitrary, but also the clinical reality: COPD, type 2 diabetes, and depression/anxiety all have an insidious onset.

Since the outcome diseases were identified using the Patient Register, we may have missed patients diagnosed and treated in primary care. However, this would be equal for both the exposed and the unexposed group. This may be of particular concern for peripheral arterial disease, atrial fibrillation, and chronic heart failure, as in Sweden they often are diagnosed and managed in primary care, but our study would have identified more severe manifestations of the outcome diseases. For the diagnosis of COPD, we have no information about whether the diagnoses were verified by spirometry.

## Conclusions

Type 2 diabetes in combination with COPD is associated with increased risk of mortality and chronic heart failure, peripheral arterial disease, and atrial fibrillation, at a greater magnitude than would be expected from the sum of risks associated with the individual diseases. Depression/anxiety in combination with COPD is associated with increased risk of mortality, cerebrovascular disease, chronic heart failure, ischemic heart disease, and peripheral arterial disease. These results emphasise the importance of clinicians anticipating the disproportionally raised risk for some outcomes associated with comorbid type 2 diabetes or depression/anxiety in COPD patients. Further research should investigate whether management strategies, particularly in primary care, should be tailored to tackle these specific disease combinations.

## Electronic supplementary material

Below is the link to the electronic supplementary material.

Supplementary Material 1

Supplementary Material 2

## Data Availability

The data underlying this study cannot be shared publicly due to regulations under the relevant Swedish laws. Researchers who are interested in Swedish register data can refer to http://www.registerforskning.se/en/. Inquiries about the data used for this study can be addressed to the corresponding author.

## Abbreviations

CI: Confidence intervals
COPD: Chronic obstructive pulmonary disease
DDD: Defined daily dose
HR: Hazard ratios
LAMA: Long-acting muscarinic antagonists
RERI: Relative excess risk due to interaction
