# Supplementary material for: Disproportionately raised risk of adverse outcomes in patients with COPD and comorbid type 2 diabetes or depression: Swedish register-based cohort study
Source: Respir Res. 2025 Mar 5;26:84. doi: 10.1186/s12931-025-03160-6 (PMC11883964; doi:10.1186/s12931-025-03160-6)
Supplement: Supplementary file 1 — Supplementary Material 1 [file 12931_2025_3160_MOESM1_ESM.docx]

Supplemental Table 1 COPD and type 2 diabetes, sensitivity analysis

| **Outcome** | **No of outcome events/**  **individuals** | **Adjusted** | |
| --- | --- | --- | --- |
|  | **n** | **HR (95% CI)** | **Additive interaction**  **(95% CI)** |
| ***Cardiovascular disease*** |  |  |  |
| **Atrial fibrillation** | 420 085/  5 322 229 |  |  |
| No diabetes or COPD |  | Reference | 0.08 (0.01, 0.15) |
| Type 2 diabetes |  | 1.35 (1.34, 1.36) |  |
| COPD |  | 1.55 (1.53, 1.58) |  |
| COPD and type 2 diabetes |  | 1.98 (1.91, 2.05) |  |
| **Cerebrovascular disease** | 334 391/  5 334 593 |  |  |
| No diabetes or COPD |  | Reference | -0.09 (-0.16, -0.02) |
| Type 2 diabetes |  | 1.48 (1.46, 1.49) |  |
| COPD |  | 1.30 (1.27, 1.32) |  |
| COPD and type 2 diabetes |  | 1.68 (1.62, 1.75) |  |
| **Chronic heart failure** | 339 012/  5 376 513 |  |  |
| No diabetes or COPD |  | Reference | 0.65 (0.53, 0.77) |
| Type 2 diabetes |  | 1.86 (1.84, 1.87) |  |
| COPD |  | 2.56 (2.52, 2.60) |  |
| COPD and type 2 diabetes |  | 4.06 (3.95, 4.18) |  |
| **Ischemic heart disease** | 398 205/  5 225 566 |  |  |
| No diabetes or COPD |  | Reference | -0.04 (-0.13, 0.05) |
| Type 2 diabetes |  | 1.68 (1.67, 1.70) |  |
| COPD |  | 1.70 (1.67, 1.73) |  |
| COPD and type 2 diabetes |  | 2.34 (2.26, 2.42) |  |
| **Peripheral arterial disease** | 158 342/  5 419 304 |  |  |
| No diabetes or COPD |  | Reference | -0.01 (-0.16, 0.14) |
| Type 2 diabetes |  | 1.74 (1.72, 1.77) |  |
| COPD |  | 2.51 (2.45, 2.56) |  |
| COPD and type 2 diabetes |  | 3.24 (3.10, 3.38) |  |
|  |  |  |  |
| ***Mortality*** |  |  |  |
| **All-cause mortality** | 984 840/  5 466 424 |  |  |
| No diabetes or COPD |  | Reference | 0.19 (0.13, 0.24) |
| Type 2 diabetes |  | 1.70 (1.69, 1.71) |  |
| COPD |  | 2.06 (2.04, 2.07) |  |
| COPD and type 2 diabetes |  | 2.94 (2.89, 3.00) |  |
| **Cardiovascular death** | 593 607/  5 466 424 |  |  |
| No diabetes or COPD |  | Reference | 0.46 (0.39, 0.54) |
| Type 2 diabetes |  | 1.87 (1.86, 1.88) |  |
| COPD |  | 2.04 (2.01, 2.06) |  |
| COPD and type 2 diabetes |  | 3.37 (3.30, 3.45) |  |
| **Respiratory death** | 195 373/  5 466 424 |  |  |
| No diabetes or COPD |  | Reference | 1.17 (0.96, 1.38) |
| Type 2 diabetes |  | 1.61 (1.59, 1,63) |  |
| COPD |  | 6.03 (5.95, 6.11) |  |
| COPD and type 2 diabetes |  | 7.81 (7.61, 8.01) |  |
| COPD diagnosis first identified by the Drug Register. Cox regressions with COPD and type 2 diabetes diagnoses being modelled as time-varying covariates. The adjusted HRs were obtained by controlling for sex, highest attained level of education, county of residence, and entrance year in the study. The additive interactions were calculated as the relative excess risk due to interaction (RERI).  Abbreviations: HR hazard ratio; CI confidence interval | | | |
