# Supplementary material for: Disproportionately raised risk of adverse outcomes in patients with COPD and comorbid type 2 diabetes or depression: Swedish register-based cohort study
Source: Respir Res. 2025 Mar 5;26:84. doi: 10.1186/s12931-025-03160-6 (PMC11883964; doi:10.1186/s12931-025-03160-6)
Supplement: Supplementary file 2 — Supplementary Material 2 [file 12931_2025_3160_MOESM2_ESM.docx]

Supplemental Table 2 COPD and depression/anxiety, sensitivity analysis

| **Outcome** | **No of outcome events/**  **individuals** | **Adjusted** | |
| --- | --- | --- | --- |
|  | **n** | **HR (95% CI)** | **Additive interaction**  **(95% CI)** |
| ***Cardiovascular disease*** |  |  |  |
| **Atrial fibrillation** | 420 085/  5 322 229 |  |  |
| No COPD or depression/anxiety |  | Reference | <-0.01 (-0.05, 0.05) |
| Depression/anxiety |  | 1.04 (1.03, 1.04) |  |
| COPD |  | 1.54 (1.52, 1.57) |  |
| COPD and depression/anxiety |  | 1.58 (1.54, 1.62) |  |
| **Cerebrovascular disease** | 334 391/  5 334 593 |  |  |
| No COPD or depression/anxiety |  | Reference | 0.06 (0.01, 0.11) |
| Depression/anxiety |  | 1.36 (1.35, 1.37) |  |
| COPD |  | 1.23 (1.20, 1.26) |  |
| COPD and depression/anxiety |  | 1.65 (1.61, 1.70) |  |
| **Chronic heart failure** | 339 012/  5 376 513 |  |  |
| No COPD or depression/anxiety |  | Reference | 0.38 (0.31, 0.45) |
| Depression/anxiety |  | 1.29 (1.28, 1.30) |  |
| COPD |  | 2.42 (2.38, 2.46) |  |
| COPD and depression/anxiety |  | 3.10 (3.03, 3.16) |  |
| **Ischemic heart disease** | 398 205/  5 225 566 |  |  |
| No COPD or depression/anxiety |  | Reference | 0.12 (0.06, 0.18) |
| Depression/anxiety |  | 1.19 (1.18, 1.20) |  |
| COPD |  | 1.61 (1.58, 1.64) |  |
| COPD and depression/anxiety |  | 1.93 (1.88, 1.97) |  |
| **Peripheral arterial disease** | 158 342/  5 419 304 |  |  |
| No COPD or depression/anxiety |  | Reference | 0.20 (0.09, 0.30) |
| Depression/anxiety |  | 1.32 (1.31, 1.34) |  |
| COPD |  | 2.37 (2.31, 2.43) |  |
| COPD and depression/anxiety |  | 2.89 (2.80, 2.98) |  |
|  |  |  |  |
| ***Mortality*** |  |  |  |
| **All-cause mortality** | 984 840/  5 466 424 |  |  |
| No COPD or depression/anxiety |  | Reference | 0.25 (0.20, 0.30) |
| Depression/anxiety |  | 2.38 (2.37, 2.39) |  |
| COPD |  | 2.07 (2.05, 2.09) |  |
| COPD and depression/anxiety |  | 3.70 (3.66, 3.74) |  |
| **Cardiovascular death** | 593 607/  5 466 424 |  |  |
| No COPD or depression/anxiety |  | Reference | 0.33 (0.27, 0.39) |
| Depression/anxiety |  | 2.27 (2.26, 2.28) |  |
| COPD |  | 2.04 (2.01, 2.07) |  |
| COPD and depression/anxiety |  | 3.63 (3.58, 3.69) |  |
| **Respiratory death** | 195 373/  5 466 424 |  |  |
| No COPD or depression/anxiety |  | Reference | 3.98 (3.78, 4.18) |
| Depression/anxiety |  | 2.64 (2.62, 2.67) |  |
| COPD |  | 6.07 (5.97, 6.17) |  |
| COPD and depression/anxiety |  | 11.70 (11.51, 11.89) |  |
| COPD diagnosis first identified by the Drug Register. Cox regressions with COPD and depression/anxiety diagnoses being modelled as time-varying covariates. The adjusted HRs were obtained by controlling for sex, highest attained level of education, county of residence, and entrance year in the study. The additive interactions were calculated as the relative excess risk due to interaction (RERI).  Abbreviations: HR hazard ratio; CI confidence interval | | | |
